# Supplementary material for: Assessing the role of collectivism and individualism on COVID-19 beliefs and behaviors in the Southeastern United States
Source: PLoS One. 2023 Jan 20;18(1):e0278929. doi: 10.1371/journal.pone.0278929 (PMC9858878; doi:10.1371/journal.pone.0278929)
Supplement: S1 File — Qualitative Information Guide. (PDF) [file pone.0278929.s001.pdf]

Supporting Information 1 – Interview Guide

QUALITATIVE INTERVIEW GUIDE

Introduction: [information sheet review, study aims, goals – state that for this interview, we are interested in beliefs and attitudes of yours and the larger society]

Demographic Questions:

Age in years at time of interview: ___________ o Prefer not to say

Gender

o Male o Female o Non-binary o Transgender o Other gender o Prefer not to say

Race/Ethnicity

o African American/Black o American Indian or Alaska Native o Asian o Caucasian/White o Native Hawaiian or other Pacific Islander o Other race o More than one race o Prefer not to say.

Are you Hispanic? o Yes o No o Prefer not to say

Highest Level of Education

o Less than high school o High school graduate/GED o Some college o Associate degree o Bachelor's degree o Master's degree o Doctorate (PhD) o Professional degree (e.g., MD or JD). o Prefer not to say

Total Household Income

o Less than $10K o$10K to $19,999 o$20K to $29,999K o$30K to $39,999

o $40K to $49,999 o$50,000k to $59,999 o$60,000 to $69,999 o$70K to $79,999

o $80K to $89,999 o $90K to $99,999 o$100K to $149,999 o $150K or more

o Prefer not to say

FSU Subject Category

o Faculty o Staff o Student, undergraduate o Student, graduate or professional

Political Affiliation

o Democrat o Republican o Independent o Other party, specify: ___________________________ o No specific political affiliation o Prefer not to say

Current Religious Affiliation

o Christian religions (e.g., Catholic, Protestant) o Jewish o Muslim o Sikh o Hindu

o Buddhist o Atheist (do not believe in God) o Agnostic (not sure if there is a God)
o Other religion, specify: ________________________________) o No specific religious affiliation
o Prefer not to say

Opening Questions:

Let's start with a broad, opening question talking about the COVID-19 pandemic in general. In what ways has the pandemic impacted your life?

Can you walk me through a typical weekday in your life after COVID-19 hit the U.S.? What are the most significant differences in your daily life now compared to before the pandemic?

Semi-structured Questions:

What precautions do you take to prevent spread? (mask wearing, handwashing etc.) Why do you/or why do you not practice these behaviors? Why do you think others do/do not practice these behaviors?

What are your feelings about the COVID-19 vaccinations? Will you take/have you taken the COVID-19 vaccine when it becomes available to you? Why or why not

Compare the US to other countries regarding handling the pandemic. What are similarities, differences? What are your views on the U.S. response?

Do you think individuals, communities, society, government should be things differently? How should things be done differently?

How do you think our culture/society affects the way people in the U.S. have been reacting to the pandemic?

How do you think COVID-19 has impacted U.S. Culture and belief systems? How have your belief systems been impacted?

How do you think U.S. culture and society will be different after COVID-19?
