# Supplementary material for: Assessing the role of collectivism and individualism on COVID-19 beliefs and behaviors in the Southeastern United States
Source: PLoS One. 2023 Jan 20;18(1):e0278929. doi: 10.1371/journal.pone.0278929 (PMC9858878; doi:10.1371/journal.pone.0278929)
Supplement: S2 File — Available online at https://osf.io/vp3ke/ (PDF) [file pone.0278929.s002.pdf]

Supporting Information 2 – Quantitative Survey

Available online at https://osf.io/vp3ke/
