# Supplementary material for: Assessing the role of collectivism and individualism on COVID-19 beliefs and behaviors in the Southeastern United States
Source: PLoS One. 2023 Jan 20;18(1):e0278929. doi: 10.1371/journal.pone.0278929 (PMC9858878; doi:10.1371/journal.pone.0278929)
Supplement: S3 File — Evaluation tool for collectivism/individualism. (PDF) [file pone.0278929.s003.pdf]

Supporting Information 3

CULTURE ORIENTATION SCALE (COS) Questions (adapted from Triandis and Gelfand 1998**)**

1) I'd rather depend on myself than others.

- o Strongly disagree (1)
- o Disagree (2)
- o Moderately disagree (3)
- o Mildly disagree (4)
- o Neither agree nor disagree (5)
- o Mildly agree (6)
- o Moderately agree (7)
- o Agree (8)
- o Strongly agree (9)

2) I rely on myself most of the time; I rarely rely on others.

- o Strongly disagree (1)
- o Disagree (2)
- o Moderately disagree (3)
- o Mildly disagree (4)
- o Neither agree nor disagree (5)
- o Mildly agree (6)
- o Moderately agree (7)
- o Agree (8)
- o Strongly agree (9)

3) I often do "my own thing."

- o Strongly disagree (1)
- o Disagree (2)
- o Moderately disagree (3)
- o Mildly disagree (4)
- o Neither agree nor disagree (5)
- o Mildly agree (6)
- o Moderately agree (7)
- o Agree (8)
- o Strongly agree (9)

4) My personal identity, independent of others, is very important to me.

- o Strongly disagree (1)
- o Disagree (2)
- o Moderately disagree (3)
- o Mildly disagree (4)
- o Neither agree nor disagree (5)
- o Mildly agree (6)
- o Moderately agree (7)
- o Agree (8)
- o Strongly agree (9)

5) If a coworker gets a prize, I would feel proud.
- o Strongly disagree (1)
- o Disagree (2)
- o Moderately disagree (3)
- o Mildly disagree (4)
- o Neither agree nor disagree (5)
- o Mildly agree (6)
- o Moderately agree (7)
- o Agree (8)
- o Strongly agree (9)

6) The well-being of my coworkers is important to me.
- o Strongly disagree (1)
- o Disagree (2)
- o Moderately disagree (3)
- o Mildly disagree (4)
- o Neither agree nor disagree (5)
- o Mildly agree (6)
- o Moderately agree (7)
- o Agree (8)
- o Strongly agree (9)

7) To me, pleasure is spending time with others.
- o Strongly disagree (1)
- o Disagree (2)
- o Moderately disagree (3)
- o Mildly disagree (4)
- o Neither agree nor disagree (5)
- o Mildly agree (6)
- o Moderately agree (7)
- o Agree (8)
- o Strongly agree (9)

8) I feel good when I cooperate with others.
- o Strongly disagree (1)
- o Disagree (2)
- o Moderately disagree (3)
- o Mildly disagree (4)
- o Neither agree nor disagree (5)
- o Mildly agree (6)
- o Moderately agree (7)
- o Agree (8)
- o Strongly agree (9)

9) It is important that I do my job better than others.
- o Strongly disagree (1)
- o Disagree (2)
- o Moderately disagree (3)

- o Mildly disagree (4)
- o Neither agree nor disagree (5)
- o Mildly agree (6)
- o Moderately agree (7)
- o Agree (8)
- o Strongly agree (9)

10) Winning is everything.

- o Strongly disagree (1)
- o Disagree (2)
- o Moderately disagree (3)
- o Mildly disagree (4)
- o Neither agree nor disagree (5)
- o Mildly agree (6)
- o Moderately agree (7)
- o Agree (8)
- o Strongly agree (9)

11) Competition is the law of nature.

- o Strongly disagree (1)
- o Disagree (2)
- o Moderately disagree (3)
- o Mildly disagree (4)
- o Neither agree nor disagree (5)
- o Mildly agree (6)
- o Moderately agree (7)
- o Agree (8)
- o Strongly agree (9)

12) When another person does better than I do, I get tense and aroused.

- o Strongly disagree (1)
- o Disagree (2)
- o Moderately disagree (3)
- o Mildly disagree (4)
- o Neither agree nor disagree (5)
- o Mildly agree (6)
- o Moderately agree (7)
- o Agree (8)
- o Strongly agree (9)

13) Parents and children must stay together as much as possible.

- o Strongly disagree (1)
- o Disagree (2)
- o Moderately disagree (3)
- o Mildly disagree (4)

- o Neither agree nor disagree (5)
- o Mildly agree (6)
- o Moderately agree (7)
- o Agree (8)
- o Strongly agree (9)

14) It is my duty to take care of the family, even when I have to sacrifice what I want.

- o Strongly disagree (1)
- o Disagree (2)
- o Moderately disagree (3)
- o Mildly disagree (4)
- o Neither agree nor disagree (5)
- o Mildly agree (6)
- o Moderately agree (7)
- o Agree (8)
- o Strongly agree (9)

15) Family members should stick together, no matter what sacrifices are required.

- o Strongly disagree (1)
- o Disagree (2)
- o Moderately disagree (3)
- o Mildly disagree (4)
- o Neither agree nor disagree (5)
- o Mildly agree (6)
- o Moderately agree (7)
- o Agree (8)
- o Strongly agree (9)

16) It is important to me that I respect the decisions made by my groups.

- o Strongly disagree (1)
- o Disagree (2)
- o Moderately disagree (3)
- o Mildly disagree (4)
- o Neither agree nor disagree (5)
- o Mildly agree (6)
- o Moderately agree (7)
- o Agree (8)
- o Strongly agree (9)

End of Block: Individualism vs. Collectivism
