# Supplementary material for: Assessing the role of collectivism and individualism on COVID-19 beliefs and behaviors in the Southeastern United States
Source: PLoS One. 2023 Jan 20;18(1):e0278929. doi: 10.1371/journal.pone.0278929 (PMC9858878; doi:10.1371/journal.pone.0278929)
Supplement: S4 File — Comparison of Results from Traditional Logistic Regression and Those adjusted for Rare Events Using the Firth Method. (PDF) [file pone.0278929.s004.pdf]

Supporting Information 4. Comparison of Results from Traditional Logistic Regression and Those adjusted for Rare Events Using the Firth Method.

Logistic Regression on being Vaccinated and Unvaccinated by Collectivism (N=251).

**Outcome**	Standard Logistic Regression (Table 3 Results) **Vaccinated**			Adjusted using Firth Method **Unvaccinated**		
Covariate	Model 1	Model 2	Model 3	Model 1	Model 2	Model 3
Collectivism	0.010<br>(0.022)	-0.003<br>(0.025)	-0.003<br>(0.026)	-0.009<br>(0.022)	0.003<br>(0.024)	0.003<br>(0.024)
Age	0.030<br>(0.024)	0.031<br>(0.029)	0.031<br>(0.030)	-0.029<br>(0.023)	-0.029<br>(0.029)	-0.029<br>(0.028)
High Risk	0.348<br>(0.793)	1.253<br>(1.015)	1.252<br>(.016)	-0.226<br>(0.756)	-1.065<br>(0.976)	-1.048<br>(0.971)
Republican		-3.226***<br>(0.627)	-3.227***<br>(0.635)		3.053***<br>(0.594)	3.018***<br>(0.596)
Female			-0.009<br>(0.620)			-0.024<br>(0.590)
Constant	0.452<br>(1.942)	2.229<br>(2.211)	2.228<br>(2.211)	-0.451<br>(1.916)	-2.164<br>(2.144)	-2.131<br>(2.135)

Standard errors are in parentheses. _Constant represents the Y intercept.
+p<.10; **p* < .05; ***p* < .01; ****p* < .001.

The results in the above appendix table indicate that the key findings of the study do not differ across models using standard logistic regression and those that use the Firth Method to adjust for rare events (in this case being unvaccinated was rare in our sample). The key findings are that collectivism is unrelated to the probability of vaccination, and being a republican is a strong predictor of being unvaccinated. The data are consistent regardless of the analytic approach.
