# Supplementary material for: Assessing the role of collectivism and individualism on COVID-19 beliefs and behaviors in the Southeastern United States
Source: PLoS One. 2023 Jan 20;18(1):e0278929. doi: 10.1371/journal.pone.0278929 (PMC9858878; doi:10.1371/journal.pone.0278929)
Supplement: Attachment — Submitted filename: JMM Revisions to Covid PLOS.docx [file pone.0278929.s005.docx]

Dear Dr. Abbas Al Mutair, Academic Editor of PLOS ONE,

We are excited to re-submit our revised article for publication with PLOS ONE. As reviewers noted, this article presents an interesting perspective on collectivism/individualism and safe COVID-19 behaviors on an American college campus.

We conducted interviews and delivered a quantitative survey on Florida State University’s campus, assessing perspectives on individualism and collectivism. We evaluated these viewpoints relative to COVID-19 behaviors and practices. We found that collectivism is correlated with increasing age and education, and that collectivism is correlated with safe COVID-19 behaviors. Increased consumption of conservative news media is correlated with disregard of safe COVID-19 behaviors. Our survey sample of 3000 comes from the FSU campus population of faculty, staff, and students.

In our revised manuscript, we have made every effort to conform to PLOS ONE guidelines and to address concerns from Reviewer 1 and Reviewer 2. Our efforts are outlined in this document and the changes made to the document are substantial. We thank the reviewers for their comments.

Please feel free to reach out to us if you have any questions or concerns. We look forward to working with you towards the publication of this new and exciting work.

Kindest regards,

Jayur Mehta, Assistant Professor, Anthropology, Florida State University

Choeeta Chakrabarti, Specialized Teaching Faculty, Anthropology, Florida State University

Jessica De Leon, Assistant Professor and Associate in Research, College of Medicine, Florida State University

Patricia Homan, Assistant Professor of Sociology and Associate Director of the Public Health

Program, Florida State University

Tara Skipton, Graduate Student, Anthropology, University of Texas Austin

Rachel Sparkman, Graduate Student, Sociology, Florida State University

**OVERALL REVISION COMMENTS**

**JMM et al Revisions to Covid-19 Article for PLOS**

1. Please ensure that your manuscript meets PLOS ONE's style requirements, including those for file naming. The PLOS ONE style templates can be found at 
[https://journals.plos.org/plosone/s/file?id=wjVg/PLOSOne_formatting_sample_main_body.pdf](https://urldefense.com/v3/__https:/journals.plos.org/plosone/s/file?id=wjVg*PLOSOne_formatting_sample_main_body.pdf__;Lw!!PhOWcWs!3Cf9Ek4knRph8FBUh-45jo2zBg-nKxqfesz3OPKGsnyzAEuZ9Ro7hPrbHkta9ahoCmb7fpe-lQEP6nE$) and 
[https://journals.plos.org/plosone/s/file?id=ba62/PLOSOne_formatting_sample_title_authors_affiliations.pdf](https://urldefense.com/v3/__https:/journals.plos.org/plosone/s/file?id=ba62*PLOSOne_formatting_sample_title_authors_affiliations.pdf__;Lw!!PhOWcWs!3Cf9Ek4knRph8FBUh-45jo2zBg-nKxqfesz3OPKGsnyzAEuZ9Ro7hPrbHkta9ahoCmb7fpe-h7A-YKM$)

**JMM RESPONSE**

**-** *yes, we have reviewed the style templates and have revised our file accordingly.*

2. We note that you have stated that you will provide repository information for your data at acceptance. Should your manuscript be accepted for publication, we will hold it until you provide the relevant accession numbers or DOIs necessary to access your data. If you wish to make changes to your Data Availability statement, please describe these changes in your cover letter and we will update your Data Availability statement to reflect the information you provide.

**JMM RESPONSE**

- *We have placed the data and code on OSF with the following permanent link:*

- <https://osf.io/vp3ke/>

**Comments to the Author**

1. Is the manuscript technically sound, and do the data support the conclusions?

The manuscript must describe a technically sound piece of scientific research with data that supports the conclusions. Experiments must have been conducted rigorously, with appropriate controls, replication, and sample sizes. The conclusions must be drawn appropriately based on the data presented.

Reviewer #1: Partly

Reviewer #2: Yes

**NO RESPOSE REQUIRED HERE**

2. Has the statistical analysis been performed appropriately and rigorously?

Reviewer #1: Yes

Reviewer #2: Yes

**NO RESPOSE REQUIRED HERE**

3. Have the authors made all data underlying the findings in their manuscript fully available?

The [PLOS Data policy](https://urldefense.com/v3/__http:/www.plosone.org/static/policies.action*sharing__;Iw!!PhOWcWs!3Cf9Ek4knRph8FBUh-45jo2zBg-nKxqfesz3OPKGsnyzAEuZ9Ro7hPrbHkta9ahoCmb7fpe-r24Vxww$) requires authors to make all data underlying the findings described in their manuscript fully available without restriction, with rare exception (please refer to the Data Availability Statement in the manuscript PDF file). The data should be provided as part of the manuscript or its supporting information, or deposited to a public repository. For example, in addition to summary statistics, the data points behind means, medians and variance measures should be available. If there are restrictions on publicly sharing data—e.g. participant privacy or use of data from a third party—those must be specified.

Reviewer #1: No
Reviewer #2: No

**Response: We have placed the data and code on OSF with the following permanent link:** [**https://osf.io/vp3ke/**](https://osf.io/vp3ke/)

5. Review Comments to the Author

Reviewer #1: This article provides an interesting perspective into the challenges and experiences of Americans during the COVID-19 pandemic. The manuscript explores perceptions of behaviors and attitudes that how certain collective or individualistic orientations might foster less or more mask use. This paper has potential but needs major revision to be considered.


Authors should post their Data and code on OSF with a permanent link. Not a wordpress site.

**Response: We have placed the data and code on OSF with the following permanent link:** [**https://osf.io/vp3ke/**](https://osf.io/vp3ke/)

Reviewer #2: Review: Assessing the role of collectivism and individualism on COVID-19 Beliefs and Behaviors

Analysis:
I am not sure whether it make sense to run a logistic regression with n=251 for vaccinated when 93% of people were vaccinated. If anything, the authors should consider a zero-inflated logistic regression model (with vacc recoded as zero).

**JMM ET AL RESPONSE**

*Response: The reviewers make an excellent point regarding the need to consider alternative analyses which account for the small number of unvaccinated cases in our sample. We therefore estimated supplemental models using the firth method to adjust for rare events (see:* [*https://www3.nd.edu/~rwilliam/stats3/rareevents.pdf*](https://www3.nd.edu/~rwilliam/stats3/rareevents.pdf)*). The results did not differ meaningfully from our original results presented in table 3 and figures 2 & 3—collectivism was still unrelated to the probability of vaccination, and being a republican was still a strong predictor of being unvaccinated. Therefore we kept our original approach for consistency across outcomes and ease of interpretation (i.e., higher probabilities are linked to more safe COVID behaviors for all outcomes), but we have included the supplemental models in Appendix 4 for comparison.*

It should be explained explicitly why age was included in Model 1 but gender in Model 3 (I think it makes sense but it should be explained).

**Response: We now include additional information about the nested models and including gender in Model 3.**

Table 3: it should be specified in the table notes what _cons means.
Table 4: it should be specified in the table notes what femalefm and _cons means.

**Response: Tables 3 and 4 have been updated for clarity.**

**INDIVIDUAL REVIEWER REVISION COMMENTS**

Reviewer 1

1) Structure of the paper is abnormal and recommendation is for the literature review to follow typical plosone articles or typical social science articles.
   a. JMM RESPONSE
      i. I reviewed several social science PlosONE articles and re-organized my paper to follow the Plos format more closely. However, some Plos articles do not have a detailed background/literature review section, and I felt this was an important part of the article. In addition, R1 wanted me to add more citations into my literature review, so I thought it was necessary to maintain my literature review in its current position.
      ii. I also removed the section *WHAT IS AN AMERICAN IDENTITY, which did not directly tie in to the article.*
2) Add necessary citations to literature review .
   a. JMM RESPONSE
      i. I made sure to include the recommended citations that RI suggested I cite.
         1. *Added Lu et al 2021 PNAS, English and Li 2021 and English et al 2022*.
3) Weak point in methodology b/c only 283 participants responded out of 3000 polled
   a. JMM RESPONSE
      i. Most of the survey team were fairly happy with the survey response given no incentives were provided. We had expected approximately 10% response rate and we received just below a 10% response rate.
      ii. We believe the 10% response rate can be attributed to the length of the survey (over 15 minutes) and that most individuals were working remotely and overwhelmed by zoom and by the amount of time they were spending on their computer. Unfortunately, this is nothing that we can change at the moment.
          1. I added a statement about the response rate in the RESULTS section
             a. *While a higher response rate would be desirable, our response rates are consistent with (and in many cases higerh than) what is typical for online studies which have recently published results in peer-reviewed scientific outlets [51–53]. It is likely that the mental exhaustion of most individuals during the COVID-19 pandemic, and the overwhelming amount of time individuals spent on their computers, may have lowered the response rate of this survey, but it is not clear how this would affect the results.*
4) Mixed method approach unclear
   a. JMM RESPONSE
      i. We do have some language explaining mixed methods and how interviews and qualitative data influenced our quantitative data collection but I added more text to make it clearer.
         1. I added this line
            a. *The purpose of Phase 1 and this mixed-methods approach was to use respondent answers and ideas to build and design a more purposeful and insightful survey questionnaire.*
5) Reconsider interpretation given the study population is from FSU and that Florida had a unique path in the pandemic. Reframe conclusion and interpretation with caution for local context. R1 recommends further analysis to explain where people are from and how it may play an interactive effect on attitudes.
   a. JMM RESPONSE
      i. Unfortunately, we cannot trace where our respondent are/were from, as that question was not included within the quantitative survey. We have some demographic data but not where they are from or where they live presently. I agree with R1 that this presents a fascinating opportunity to evaluate an urban/rural divide in terms of COVID-19 practices, but it is not within the capacity of the dataset we have presently.
      ii. I note R1’s concern about the study population and I make note in my discussion at the end about how the study sample impacts our analysis. I added the following text in the **Discussion** section.
          1. *Finally, in terms of our study population, we should not think of our sample as a heavily skewed set of professors. Many of the faculty, staff, and students that work at FSU come from a wide-ranging territory in the surrounding counties and from across Florida, which can be variously conservative and/or liberal. Only 58% of our sample respondents self-identified as Democrat. The study sample is not ethnically diverse and skews white, well-educated, and more affluent than the national average. Our respondents were also neither overwhelmingly collectivist nor were they highly individualistic. As indicated above, our sample average is just slightly more to the collectivist side of center/neutral. Future studies should strive to identify a greater diversity of respondents, where they were raised and where they live presently, and how their background influences collectivism and health safety practices. While we cannot use this study and/or this study population to make grand claims about the US population writ large, we do have the ability to discuss fascinating patterns in the data. In particular, what factors influenced our 93% vaccination rate? Furthermore, whether collectivist or individualist, more than half (73%) reported wearing masks, indicating that consistent and pervasive health policy messaging was positively impacting safe COVID-19 behaviors. Understanding why is still more challenging yet, and additional studies are needed to elucidate these fascinating trends.*
      iii. I also added text in the **Synthesis and Future Outlook** section
           1. *It is likely that the politicization of the pandemic in its early days played a significant role in how individuals perceived their efficacy of social isolation, mask-wearing, and vaccines. Variable and disjointed policies and lack of enforcement certainly may have contributed to our excessive mortality rates[17], likely also influenced by uniquely American patterns of health [66–69]. The rural and urban divide certainly impacted mask-wearing and mask prevalence in parts of China, where mask-wearing was variously adopted according to government measure, population density, and community practice [4,70,71]. How might we understand differences in rural and urban health practices and COVID-19 outcomes in the United States; numerous studies have indicated that rurality impacts safe COVID-19 practice due to increased age, political ideology, and community experiences tied to population density [72–74]? Our study demonstrates practices of a relatively urban population surrounded by a rural community, and future studies in North Florida should seek to disentangle rural/urban practices and their relationships to individualism and collectivism.*
6) Authors should post their data and code on OSF
   a. Agreed, and we created an OSF project and place to store code and data.
      i. ***LINK -***

Reviewer 2

1) Both R1 and R2 that I need to dial back how my interpretations might apply broadly across America and instead focus on the local and regional implications for my data. I agree with their points and have made efforts throughout the paper to not use my data to talk broadly about the US as a whole.
   a. Most of the edits germane to R1 and R2’s concerns have been pasted above under R1.
   b. I removed the entire literature section about “AMERICAN IDENTITY” because I agree R1 and R2 and I did not want to generalize my study to the entire American population. Thus, I am trying to make this article more about the local study and make it clear to the reader that I cannot make broad comparisons to the US population.
2) R2 asks to mention study limitations in the introduction.
3) R2 wonders why I present results in the introduction
   a. Moved to first paragraph of results.
4) R2 asks for a comment about small proportion of republicans
   a. I added the following –
      i. *We must however note that our sample of self-identified republicans (12%) is quite small, and future studies could strive to oversample for underrepresented categories in the study population.*
5) Like R1, R2 has some issues with the literature review.
   a. I removed the section on “AMERICAN IDENTITY” which did not seem to be so pertinent to the study.
   b. Regarding style, R2 suggests I write a background that is less discipline specific and more accessible. Also to focus on information that is relevant to the research question. R2 also has an extensive list of citations for me to include.
      i. Added R2’s citations and incorporated them into the literature review.
   c. Added a section in the LIT REVIEW called “LINKING COLLECTIVISM AND INDIVIDUALISM TO US PANDEMIC RESPONSE”
      i. This is a brand-new section and it highlights non-anthropological studies of global pandemic responses relative to individualism and collectivism.
         1. *Linking Collectivism and Individualism to U.S. Pandemic Response*
            a. *Safe COVID-19 behaviors, including mask wearing and social distancing, were practiced variously and differently across the world. Post-hoc analyses have demonstrated their effectiveness at reducing transmission and mortality [26]. In rural China, increased mask wearing was influenced by a history of rice farming, which required more cooperation and collective coordination than other forms of agriculture [27]. In other cases, collective responses to COVID-19 were structured by national policies and/or unique individual responses [27–29]. In the U.S., where the response to the pandemic was heavily politicized, mask wearing and individual responses to health safety policies were impacted by misinformation, conspiracies, and an active disdain for science [30–34], despite known links between political party affiliation and COVID-19 mortality [35,36].*
            c. *Policies emphasizing collective action and the collective good have not been the focus of U.S. development. From inaction on climate change, to crumbling national infrastructure, to disjointed COVID-19 policies, U.S. policies have favored individualism and self-fulfillment over the creation of institutions for the collective good [37,38]. Countries that were individualistic during the COVID-19 pandemic experienced greater transmission of the disease and mortality from it [39]. Studies have shown that individualist tendencies in a population result in the lack of adherence to safe COVID-19 behaviors and in greater COVID-19 transmission rates [39–42]. These studies demonstrate how individualism negatively impacts health outcomes and also demonstrate the negative impacts on politically-driven health policy and the impacts of misinformation.*
            d. *This study looks to the collective orientation scale (COS) and evaluates it against demographic data and behavioral responses elicited from our study population to understand more deeply how ideology, social organization, and personality impact local [N. Florida] pandemic responses.*
6) Fixed R2’s comments about analysis.
   a. *Insert trish and rachel’s comments here*
   b. Fixed figure captions – added captions and more text.
7) Fixed references for PLOSone style
