# Supplementary material for: Assessing the role of collectivism and individualism on COVID-19 beliefs and behaviors in the Southeastern United States
Source: PLoS One. 2023 Jan 20;18(1):e0278929. doi: 10.1371/journal.pone.0278929 (PMC9858878; doi:10.1371/journal.pone.0278929)
Supplement: Attachment — Submitted filename: JMM Responses to Reviewers ROUND 2.docx [file pone.0278929.s006.docx]

**OVERALL REVISION COMMENTS – ROUND 2 – 11/16/2022**

PONE-D-22-14363R1

Assessing the Role of Collectivism and Individualism on COVID-19 Beliefs and Behaviors

**RESPONSES TO THE REVIEWERS**

**Comments to the Author**

1. If the authors have adequately addressed your comments raised in a previous round of review and you feel that this manuscript is now acceptable for publication, you may indicate that here to bypass the “Comments to the Author” section, enter your conflict of interest statement in the “Confidential to Editor” section, and submit your "Accept" recommendation.

Reviewer #1: All comments have been addressed

Reviewer #2: (No Response)

2. Is the manuscript technically sound, and do the data support the conclusions?

The manuscript must describe a technically sound piece of scientific research with data that supports the conclusions. Experiments must have been conducted rigorously, with appropriate controls, replication, and sample sizes. The conclusions must be drawn appropriately based on the data presented.

Reviewer #1: Yes

Reviewer #2: Yes

3. Has the statistical analysis been performed appropriately and rigorously?

Reviewer #1: Yes

Reviewer #2: Yes

4. Have the authors made all data underlying the findings in their manuscript fully available?

The [PLOS Data policy](https://urldefense.com/v3/__http:/www.plosone.org/static/policies.action*sharing__;Iw!!PhOWcWs!19TTFnp1YmV4UPn_unsf-8yZCuEHFvmq2JTn7zmhn2GB_mAJU5kaQ-gySLJAGGzaeemMtJmh1D1HX70$) requires authors to make all data underlying the findings described in their manuscript fully available without restriction, with rare exception (please refer to the Data Availability Statement in the manuscript PDF file). The data should be provided as part of the manuscript or its supporting information, or deposited to a public repository. For example, in addition to summary statistics, the data points behind means, medians and variance measures should be available. If there are restrictions on publicly sharing data—e.g. participant privacy or use of data from a third party—those must be specified.

Reviewer #1: Yes

Reviewer #2: Yes

5. Is the manuscript presented in an intelligible fashion and written in standard English?

PLOS ONE does not copyedit accepted manuscripts, so the language in submitted articles must be clear, correct, and unambiguous. Any typographical or grammatical errors should be corrected at revision, so please note any specific errors here.

Reviewer #1: Yes

Reviewer #2: Yes

6. Review Comments to the Author

Please use the space provided to explain your answers to the questions above. You may also include additional comments for the author, including concerns about dual publication, research ethics, or publication ethics. (Please upload your review as an attachment if it exceeds 20,000 characters)

Reviewer #1: I support the publication of this manuscript. It has improved substantially. I would ask the authors to revise their title to into "in the Southeastern United States" because I believe a lot of key findings are very context specific to Florida. This will also be valuable for future readers who will ponder the unique COVID response in Florida as early as May 2020. It is important to note that college represents a diverse group of students and teachers who might share different opinions and attitudes from local authorities who removed mask policies at that time. It might be worth expanding on this as well.

- JMM RESPONSE
  - Added “*in the Southeastern United States*” to the end of the title.
  - R1 suggests it may be worth adding more content about how/why students and teachers may have different opinions and attitudes from local authorities.
    - I agree and add the following content:
      - *In particular, our sample consists of individuals linked to a university, whose mission is public education and research directed towards the public good. Furthermore, this university is in Leon County, a historically democratic population center, and it is likely that our sample universe embodied different values from conservative-leaning population centers in other parts of the state and country [4].*

Reviewer #2: I think the article improved greatly in the revised version.
There are still some remaining issues which should be improved:
- There is a section in the introduction with the title “Previous research on COVID-19 and the U.S. Social and Political Response”. What is listed in this four short review paragraphs is only a very small snapshot of what has been researched on this topic, and it was not clear to my how the author selected the content. I think the title of the paragraph does not match the content. The authors should state explicitly at the beginning of this section that they selected some results that they think are relevant for the present research, or the title of the paragraph should me more specific.

- JMM RESPONSE
  - Given the pace of publication on the COVID-19 pandemic, I really do believe it would be impossible to comprehensively cite all of the literature.
    - For the Problem Orientation section, and the following subsection, I changed the title from “Previous Research on COVID-19…” to “*Previous biosocial and structural research on COVID-19*…”
      - This change in the subheading makes clearer why we selected certain publications to focus on for this section. As anthropologists, we tend to focus on biosocial approaches to health.

- A formal definition of collectivism and individualism should be provided

- JMM RESPONSE
  - Agreed and added the following text:
    - *Collectivism describes a condition of society in which the needs of the group are prioritized over the individual; conversely, individualism prioritizes rights, concerns, needs, and desires of each individual [24–28]. Variations in individualism and collectivism are documented across the globe and these variations have influenced nation-state approaches to pandemic preparedness and response.*

- At the end of the introduction, the authors list some more research questions (is political affiliation related to COVID-19 safety behaviors; relationship between collectivism and trust in science). The authors should at least briefly introduce these questions with the idea behind it and the relevant references that addressed these questions already.

- JMM RESPONSE
  - I’ve read through my introduction several times, and I just do not see what R2 is referring to here… there is no list of research questions in my intro.
  - There is a list of sub-inquiries (research questions) in my METHODS section. If these are the questions they meant, I have added a sentence explaining each research question and some citations.
    - *Hui and Yee report a positive association between age and collectivism, as well as between sex (females) and collectivism [46]. Vandello and Cohen report Florida in the 2^nd^ quintile for collectivism due to the age of the population and a larger than average immigrant population [47]*
    - *Numerous studies have shown links between collectivism and safe COVID-19 behaviors [33,44,45,49,50], and we wanted to test this relationship through our sample and data.*
    - *The political consequences of the pandemic have been described in detail [22,51,52], but we wanted to know if political affiliation influenced our study sample in their responses to the pandemic.*
  - Removed the trust in science inquiry, since we do not really focus on this in the article.

- The first paragraph of the results section summarizes/discusses all relevant interpretations of the results. This is unusual, the results should be reported first.

- JMM RESPONSE
  - I might argue this is a bit of a style issue. The first paragraph of our results section summarizes all of your results first, so that way the reader has the ability to grasp the significance of the results as they read through the rest of the section. Immediately after the first paragraph summary, we provide data reporting of all our results, with context and interpretation. I think our results are clearly presented, however, if R2 really wants me to create a separate section that simple just gives numerical results of the study, I’ve typed it out below, and it can be inserted into the RESULTS section as the very first paragraph.
    - *251 cases were analyzed for collectivism, yielding an average score of 91.4 (S.D. = 11.8), which is just slightly collectivist.* *We found that age, sex, and education are associated with collectivism in our sample. We also found that collectivism predicts for mask wearing, disrupted socializing, concern for infecting others, and trust in doctors regarding COVID-19 behavior guidelines.*

- A substantial part of the discussion contains results. The structure should be improved. The discussion should start with discussing the results of the main question, not with additional results

- JMM RESPONSE
  - Our data analysis, models, and interpretation of results are all presented in the results section. What can’t be incorporated into that section are the written comments, which are qualitative, and difficult to synthesize. Consequently, we weave them into the discussion section.
  - I see R2’s point about improving structure and have revised this section, while also retaining some of the qualitative data.
  - Added –
    - *Our inquiry began with the question, how does an individual’s alignment along the collectivism/individualism scale impact their choices regarding the COVID-19 pandemic? Through a quantitative survey of FSU affiliates, we discovered age, sex, and education are associated with collectivism, and that individuals that are more collectivist also tend to wear masks, practice social distancing, have concerns for infecting others, and trust in doctors regarding COVID-19 behavior guidelines. Given our questionnaire also asked respondents questions about political affiliation, we wanted to unpack how and why political beliefs influenced our study samples, since numerous studies have shown these to be interlinked phenomena [52].*

- There should be a participant section in the method section, where one can read number and demographic information (age, gender) of the participants for the different surveys. Were there really only 11 participants who conducted the semi-structured interview? Such limitations should also be mentioned in the discussion section.

- JMM RESPONSE
  - I can see why R2 may want more data on the participants in the mixed methods survey, so I report on how many men and women we interviewed, but the interviews were not part of the analysis, only the research design to improve the survey tool.
    - *Added - (n=11 participants; 8 female, 3 male)*
  - TABLE 1, which is referenced in the RESULTS section, contains all of our demographic data for PHASE 2 Quantitative Survey Participants.
  - In the research design section, I made it clear that PHASE 1 Qualitative surveys were only used to refine the survey questionnaire, and not to interpret PHASE 2 data. Our semi-structured interviews were not really analytical components of the quantitative survey, but rather a tool we used to refine our survey instrument.
  - Added this text -
    - *Phase 1 data were not utilized in the analysis of phase 2 data, nor were respondent answers used to assist in the interpretation of phase 2 data. Rather, the semi-structured interviews were simply used as a tool to refine and improve the survey questionnaire, which is the basis for our analysis.*

7. PLOS authors have the option to publish the peer review history of their article ([what does this mean?](https://urldefense.com/v3/__https:/journals.plos.org/plosone/s/editorial-and-peer-review-process*loc-peer-review-history__;Iw!!PhOWcWs!19TTFnp1YmV4UPn_unsf-8yZCuEHFvmq2JTn7zmhn2GB_mAJU5kaQ-gySLJAGGzaeemMtJmhjXXKZHY$)). If published, this will include your full peer review and any attached files.


If you choose “no”, your identity will remain anonymous but your review may still be made public.


**Do you want your identity to be public for this peer review?** For information about this choice, including consent withdrawal, please see our [Privacy Policy](https://urldefense.com/v3/__https:/www.plos.org/privacy-policy__;!!PhOWcWs!19TTFnp1YmV4UPn_unsf-8yZCuEHFvmq2JTn7zmhn2GB_mAJU5kaQ-gySLJAGGzaeemMtJmhkL13Yvo$).

Reviewer #1: No

Reviewer #2: No
